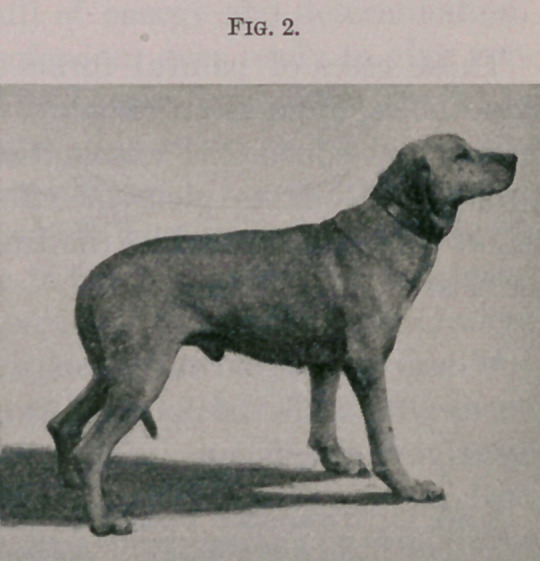# Cystoma Dermalis

**Published:** 1897-04

**Authors:** L. E. Willyoung

**Affiliations:** Buffalo, N. Y.


### CYSTOMA DERMALIS.

By L. E. Willyoung, D.V.S.,
BUFFALO, N. Y.

Case I. *Subject:* Cross-bred mastiff and hound, three years of age. *History:* Was unobtainable in detail, but swelling was present for over a year; opened repeatedly, always refilling again.

Entered at hospital February 1, 1897. Examination revealed a soft pendulous mass devoid of hair, with numerous old cicatrices on surface. Mass measuring over sixteen inches in circumference at its largest part, with a base attachment on the posterior aspect of the ulna measuring three and a half inches.

Fig. 1. Fig. 2.

On February 4th the dog was prepared for operation: a 5 per cent. solution of eucaine (methyl-ester) was injected hypodermatically and the mass removed, after making an elliptical incision on each side of the mass, vessels ligated, and skin sutured with gut. Borated gauze and iodoform dressing applied.

Upon dissection of cyst it was found to contain thirty-seven ounces of albuminous, straw-colored fluid, of alkaline reaction. Interior of cyst having numerous distinct cavities, walls of which consisted of fibro-cellular tissue, with endothelial lining. Weight of entire mass two and a half pounds.

Fig. 1 represents before operation. Fig. 2 fourteen days after operation, cicatrization nearly complete.

Eucaine was found to be an admirable substitute for cocaine, as no after-effects are noted from its use similar to the latter, while it is cheap and efficacious.

Case II. *Subject:* A spaniel puppy, six months of age. Convulsions due to intestinal irritation; persistent spasms and periods of complete coma, approaching at intervals of four to five hours. Duration five days. Elixir of bromide of potassium and chloral hydrate, with but temporary success, followed by a hypodermatic injection of $\frac{1}{20}$ grain apomorphine. Through the emesis following three plum-stones were ejected, with partial abatement of convulsions. Two days later a 2-grain hydrarg. pill was administered, when in twenty-four hours three more stones were ejected per rectum and a complete abatement of the symptoms followed.

It was found that the puppy had been fed canned plums a few days previous to the attacks.

---

Those cases of painful forms of influenza seen so frequently in sale-stables, often as an enzoötic, with great soreness of the muscles on motion, œdematous extremities painful to pressure, and with a strong tendency to pleuritic effusions, are quickly relieved and the swellings dissipated by the free use of salicylates combined with iodides.

Where corn is burnt for fuel the charred grains are found useful for mixing with the food of swine, as charcoal helps to control digestive derangements incidental to fermentation of food-products.

And still another victim to the infectious proprietary medicine mania blooms forth in Greater New York, where he hopes to emulate the father of humbugs, the late P. T. Barnum, and where he believes a confiding public is standing with open mouths and outstretched hands to praise and purchase his "nothing-ever-produced-like-them" nostrums. The flood-tide is on, the tidal-wave return is gathering, and the shores will be covered with the wrecks of these Mulberry Sellers schemes.

A so-called colored veterinary dentist suddenly disappeared from Pittsburg when he learned through a member of the State Board of Veterinary Medical Examiners that a probable wrestle in the Allegheny County courts confronted him.

Another veterinarian of Western Pennsylvania has become a victim of the proprietary medicine mania. As yet the attack is only mild, attaining a cure-all healing ointment. Only wait until the bubble bursts.

## Figures and Tables

**Fig. 1 f1:**
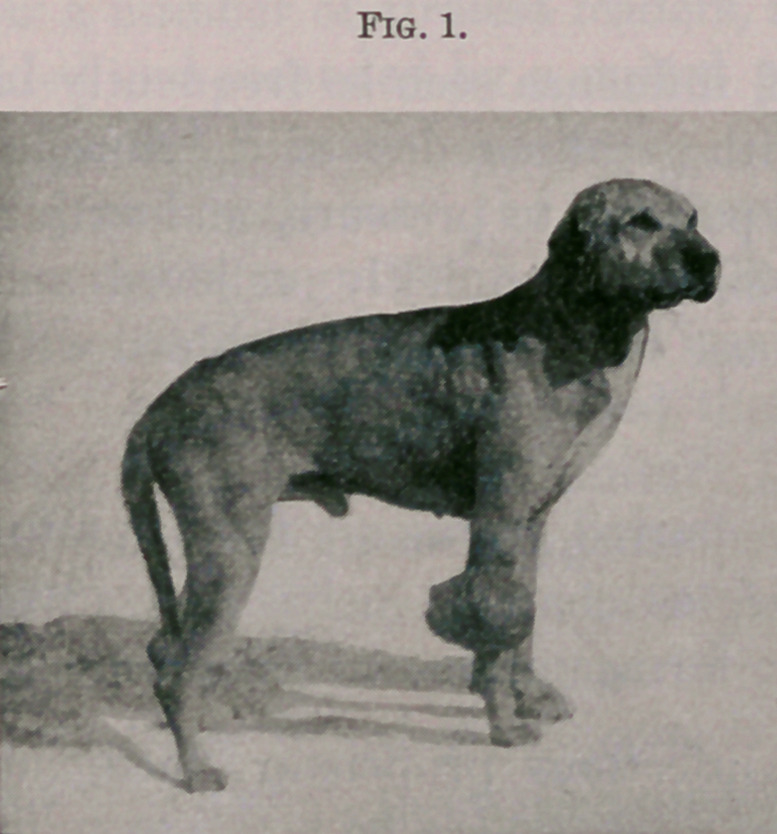


**Fig. 2. f2:**